# Disparities in Secondary Prevention of Atherosclerotic Heart Disease After Coronary Artery Bypass Grafting in Northern Plains American Indians

**DOI:** 10.1089/heq.2019.0030

**Published:** 2019-10-24

**Authors:** Hannah Kruger, Christopher Zumwalt, Rory Guenther, Rick Jansen, Donald Warne, Cornelius Dyke

**Affiliations:** ^1^ University of North Dakota School of Medicine and Health Sciences—Southeast Campus, Fargo, North Dakota.; ^2^ University of North Dakota School of Medicine and Health Sciences, Fargo, North Dakota.; ^3^ Department of Public Health, North Dakota State University, Fargo, North Dakota.; ^4^ Department of Surgery, University of North Dakota School of Medicine and Health Sciences, Fargo, North Dakota.

**Keywords:** cardiovascular disease, prevention, American Indian

## Abstract

**Introduction:** Cardiovascular disease has become the leading cause of death in American Indians (AIs). For patients with severe disease requiring coronary artery bypass grafting (CABG), AIs have been demonstrated to present with increased risk factors. Guideline-directed medical therapy after CABG effectively reduces mortality and recurrent ischemic events in all patients and is especially important in high-risk populations such as AIs.

**Methods:** Isolated CABG patients between 2012 and 2017 were studied and 74 AI patients were identified. Propensity matching was performed and the resulting 148 patients were followed for a year after surgery. Guideline-directed medical therapy (GDMT) for secondary prevention of atherosclerotic disease after CABG was detailed in all patients.

**Results:** GDMT was similar between groups (85% AI vs. 89% non-AI; *p*=NS), and the incidence of prescribed antiplatelet medications, beta-blockers, and statins was similar. AIs were more likely to receive insulin therapy (*p*=0.002) and opioids (*p*=0.03) at discharge, while non-AIs were more likely to receive anti-arrhythmic medications (*p*=0.002). One year after discharge, GDMT trended lower in AIs (75% AI vs. 85% non-AI; *p*=0.2) and AIs were less likely to be on a statin 1 year after surgery (81% AI vs. 93% non-AI; *p*=0.04). Opioid use trended higher after 1 year in AIs (28% AI vs. 18% non-AI; *p*=NS) and fewer AI patients participated in cardiac rehabilitation (CR) after CABG.

**Conclusions:** Disparities in GDMT for secondary prevention of coronary artery disease after CABG exist, with fewer AI patients receiving statins and undergoing CR 1 year after surgery. Increased use of opioids in AIs is troubling and deserves further investigation. Improved adherence to GDMT would be expected to improve long-term outcomes after CABG in this high risk population.

## Introduction

American Indians (AIs) have more cardiovascular risk factors than the general population and are at increased risk for developing cardiovascular disease (CVD).^1–5^ CVD has also become the leading cause of death in AIs.^1^ While the cause of this increase in CVD is multifactorial, disparities in socioeconomic status and societal factors contribute to this increase.^5,6^ Recent data have demonstrated that despite increased cardiovascular risk factors, Northern Plains AIs may undergo coronary artery bypass grafting (CABG) with acceptable and similar short-term outcomes as the general population.^7^ However, patients after CABG remain at risk for progression of native vessel and graft atherosclerosis and are at risk for future adverse ischemic events. Prevention of the progression of atherosclerotic heart disease after surgery is recognized as life-saving and expert consensus guidelines for the secondary prevention of coronary artery disease (CAD) exist^8^ and includes both medical and nonmedical therapies. These therapies improve survival after CABG and are recommended for all patients after coronary revascularization.^9,10^ These recommendations are especially important for vulnerable populations with increased risk factors such as AIs. Whether AI patients receive guideline-directed medical therapy (GDMT) after CABG, however, is unknown.

## Methods

Institutional Review Board approval was obtained from Sanford Health and the University of North Dakota Institutional Review Boards, and a waiver of individual consent was given due to the retrospective, chart-review nature of the study. Indian Health Service (IHS) Institutional Review Board approval was not sought as no patient contact occurred and no IHS records were utilized. Data for this single institutional study were collected retrospectively. All isolated CABG patients from June 1, 2012 to June 1, 2017 were included and 74 AI subjects were identified and propensity-matched with 74 non-AI patients, forming the two study cohorts. AIs were identified via IHSs referral or patient self-identification and patients resided both on and off federally recognized reservation land. All patients received surgery at Sanford Health Fargo and follow-up care was done at various Sanford Health hospitals and offices throughout the Upper Midwest. Patient records, medications, and other therapies were identified retrospectively in the Sanford Health electronic medical record (Epic Systems Corporation; www.EPIC.com). IHS records were not queried or utilized. Adherence to GDMT medications and other therapies were assessed at time of discharge, 4 to 6 weeks after surgery, and 1 year postoperatively. Medications at discharge reflect perioperative care and are less relevant for secondary prevention. Data at 30 days and 1 year were considered to reflect secondary prevention strategies and are reported. Adherence to GDMT was defined as taking or being prescribed antiplatelet medications, a statin, and a beta-blocker at 30 days and 1 year postoperatively. Six AI and three non-AIs were lost to follow-up at 1 year. Propensity matching between the AI and non-AI groups was done. Due to the limited sample size, the Society of Thoracic Surgeons (STS) composite risk score was used for matching. The STS composite risk score is a well-known and validated tool which incorporates multiple variables to calculate an overall individual patient risk score (http://riskcalc.sts.org/stswebriskcalc). Even after matching on this composite score, we observed differences by race for the variables sex, body mass index, and smoking. We used logistic regression to test if any of these variables were associated with any of the 1-year medications listed in Table 3 and only observed that smoking was associated with insulin (*p*=0.033), hypoglycemic (*p*=0.046), and smoking cessation (*p*=0.033).The overall lack of association between 1-year medications and these potential confounders suggests that our results are not significantly additionally influenced by these factors.

Fisher's exact tests were performed to determine significant differences between the AI and non-AI groups while controlling for time which allows us to determine if any time points were driving racial disparities. We used the MatchIt package (logit model) for the statistical program R [version 3.4.2] for propensity matching to ensure that variables were similarly distributed in our sampled population between AI and Caucasian groups. Given the limited sample size rather than choosing a specific set of variables associated with medications and race, we chose the more comprehensive STS score to match on. A *p*-value of 0.05 was considered significant.

## Results

The study population was predominantly Northern Plains Lakota, Dakota, Ojibway, and non-Hispanic whites. Preoperative clinical characteristics, patient risk factors, and operative details are detailed in Table 1. Medications at the time of discharge are listed in Table 2. The incidence of prescribed antiplatelet medication (aspirin), beta-blockers, and statin therapy was similar between AI and non-AI patients at discharge. AI patients were significantly more likely to receive insulin (*p*=0.002) and opioids (*p*=0.03) compared to non-AI patients at discharge, while non-AI patients received more antiarrhythmic medications (*p*=0.002). GDMT at discharge occurred in 89% of non-AI patients and 85% of AI patients (*p*=NS). The incidence of formal smoking cessation treatment plans was low in both groups at the time of discharge, despite higher rates of tobacco use in AIs.

**Table 1.** Patient Preoperative Characteristics

Preoperative characteristics		
Variable	American Indian % (*n*=74)	Non-American Indian % (*n*=74)
Age	60.4±11.0	68.0±9.3
Sex (female)	36.5 (27)	13.5 (10)
Height (cm)	173.6±9.4	174.7±8.4
Weight (kg)	93.1±19.3	91.6±18.1
Body mass index		
BMI category		
Underweight/normal (<24.9)	10.8 (8)	13.5 (10)
Overweight (25.0–29.9)	36.5 (27)	40.5 (30)
Obese I (30.0–34.9)	25.7 (19)	33.8 (25)
Obese II (35.0–39.9)	20.3 (15)	8.2 (6)
Morbid obese (40.0+)	6.8 (5)	4.0 (3)
Diabetes	63.5 (47)	41.9 (31)
Diabetes control		
None/diet	45.9 (34)	64.9 (48)
Oral	17.6 (13)	27.0 (20)
Insulin	36.5 (27)	8.1 (6)
Dialysis	5.4 (4)	1.4 (1)
Hypertension	89.2 (66)	78.4 (58)
Chronic lung disease	74.3 (55)	32.4 (24)
Smoker	60.8 (45)	20.3 (15)
Preop congestive heart failure	13.5 (10)	16.2 (12)
History of MI	56.8 (42)	41.9 (31)
Arrhythmia (atrial fibrillation, atrial flutter, other)	6.8 (5)	14.9 (11)

BMI, body mass index; MI, myocardial infarction.

**Table 2.** Thirty-Day Medications

Secondary prevention measures	Non-American Indian (*N*=74), *n* (%)	American Indian (*N*=71), *n* (%)	*p*
Aspirin	71 (95.9)	71 (100)	0.2451
ADP inhibitors	14 (18.9)	11 (15.5)	0.6629
Statins	69 (93.2)	61 (85.9)	0.1786
Other lipid lower medications	5 (6.76)	4 (5.63)	1.000
Beta blockers	69 (93.2)	68 (95.8)	0.7192
Other antiarrhythmics	54 (73.0)	30 (42.3)	0.0002^*^
ACEI/ARB	41 (55.4)	46 (63.8)	0.3093
Diuretics	35 (47.3)	30 (42.3)	0.6171
Insulin (NPH and analog)	15 (20.3)	32 (45.1)	0.0024^*^
Oral hypoglycemics	20 (27.0)	33 (46.5)	0.0167^*^
Other hypoglycemics	1 (1.35)	2 (2.82)	0.6146
Antidepressants	19 (25.7)	18 (25.4)	1.000
Opioids	64 (86.5)	69 (97.2)	0.0315^*^
Other pain medications	29 (39.2)	31 (43.7)	0.6159
Smoking cessation	3 (4.05)	8 (11.3)	0.1243
GDMT	66 (89.2)	61 (85.9)	0.6195

^*^ *p*<0.05.

ACEI/ARB, angiotensin converting enzyme inhibitor/angiotensin II receptor blocker; ADP, adenosine diphosphate; GDMT, guideline-directed medical therapy; NPH, neutral protamine Hagedorn.

**FIG. 1.**
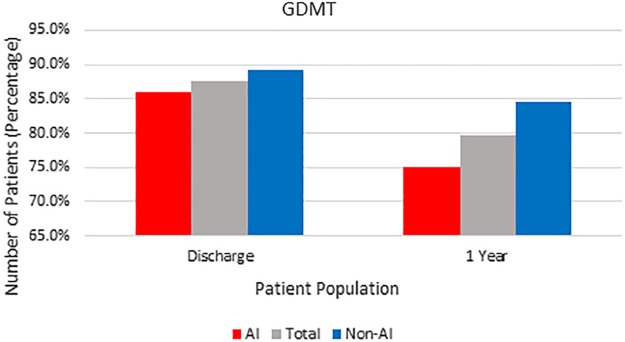
Guideline directed medical therapy after coronary artery bypass grafting.

Differences in secondary prevention were evident between groups 1 year after CABG (Table 3). Despite their higher risk, AI patients were less likely to be on a statin 1 year after CABG (non-AI 93% vs. AI 81%; *p*=0.04). Aspirin and beta-blocker usage 1 year after CABG were similar and high in both groups. The use of dual antiplatelet therapy was low in both groups at 1 year. Beta-blocker therapy was above 90% in both groups. GDMT (defined as the combination of aspirin, statin, and beta-blocker) trended lower in AI patients than non-AI patients 1 year after CABG (non-AI 85% vs. AI 75%; *p*=0.20; Fig. 1).

**Table 3.** One-Year Medications

Secondary prevention	Non-American Indian (*N*=71), *n* (%)	American Indian (*N*=68), *n* (%)	*p*
Aspirin	63 (88.7)	64 (94.1)	0.3673
ADP inhibitors	10 (14.1)	12 (17.6)	0.6451
Statins	66 (93.0)	55 (80.9)	0.0435^*^
Other lipid lower medications	3 (4.23)	4 (5.88)	0.7146
Beta blockers	65 (91.5)	62 (91.2)	1.000
Other antiarrhythmics	2 (2.82)	1 (1.47)	1.000
ACEI/ARB	25 (35.2)	39 (57.4)	0.0108^*^
Diuretics	21 (29.6)	17 (25.0)	0.5731
Insulin (NPH and analog)	12 (16.9)	29 (42.6)	0.0014^*^
Oral hypoglycemics	18 (25.4)	25 (36.8)	0.1986
Other hypoglycemics	3 (4.23)	0 (0)	0.2449
Antidepressants	12 (16.9)	12 (17.6)	1.000
Opioids	13 (18.3)	19 (27.9)	0.2272
Other pain medications	25 (35.2)	28 (41.2)	0.4897
Smoking cessation	2 (2.82)	5 (7.35)	0.2679
GDMT1	60 (84.5)	51 (75.0)	0.2054

^*^ *p*<0.05.

Other differences in prescribed medications existed 1 year after CABG. The use of ACE-inhibitors and ARBs was similar between the two groups at discharge (non-AI 55.4% vs. AI 63.8%), however, ACEI/ARB medication usage remained high in AIs while dropping significantly in non-AIs 1 year after CABG (non-AIs 35% vs. AI 57%; *p*<0.05). Insulin use was also significantly higher among AIs. Opioid usage was numerically different between AIs and non-AIs a year after CABG (non-AI 18% vs. AI 28%; *p*=0.22), although this trend was not statistically significant.

**FIG. 2.**
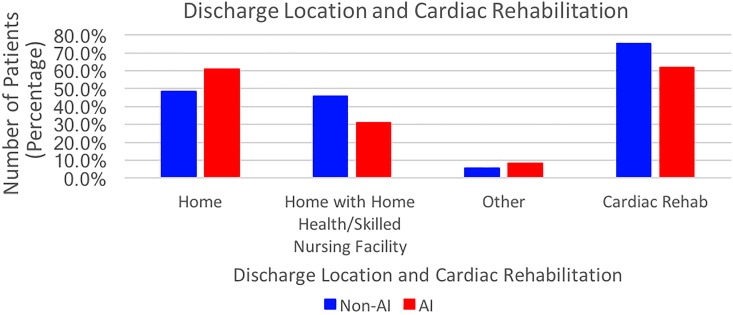
Discharge location and cardiac rehabilitation after surgery.

Non-AI subjects were more likely to be discharged to a skilled nursing facility or home with home health care compared to AI subjects, who primarily were discharged home (Fig. 2). Although not statistically significant, there appears to be a trend of fewer AIs receiving cardiac rehabilitation (CR) after CABG (75.3% non-AIs vs. 62.0% AI, *p*=NS).

## Discussion

Guideline-derived medical therapy (GDMT) decreases morbidity and mortality in patients after CABG.^8–10^ Pinho-Gomes et al. define GDMT as the use of an antiplatelet agent, a statin, and a beta-blocker after CABG, therapies known to improve graft patency, reduce cardiovascular risk, and prevent future cardiovascular events.^9^ However, initiation rates of GDMT after CABG vary and adherence to GDMT after CABG is challenging. For vulnerable populations with increased cardiovascular risk, GDMT becomes especially important. Compared to the general population, AIs are known to have increased incidence of cardiovascular risk factors such as smoking, diabetes, obesity, and hypertension.^1–4^ They are also at high risk for recurrent cardiac events after revascularization. Despite increased risk factors, CABG in AIs can be done safely, with similar short-term outcomes to the general population.^7^ Potential disparities in medium and long-term survival, however, make secondary prevention especially important. In this retrospective study, we have found that disparities in medical therapy exist after CABG with the potential to adversely impact cardiovascular health in AIs.

Adherence to GDMT for secondary prevention after coronary revascularization procedures is challenging and up to a third of patients may not receive adequate prevention as soon as 1 year after surgery.^9^ In our study, we also found that adherence to GDMT declined in both AI and non-AIs between discharge and 1 year. While not statistically significant, adherence to GDMT trended lower in AI patients (Fig. 1) and was driven by statin usage 1 year after surgery. Determining whether this disparity in GDMT is significantly different will require further evaluation with a larger patient dataset. We did find that compliance to aspirin and beta-blocker therapy in both groups stayed relatively high and consistent throughout the year, perhaps because these medications are relatively inexpensive and more readily available. Statin use was lower in the AI population 1 year after CABG (non-AI 93% vs. AI 81% *p*=0.048) and this trend toward lower statin use in AIs with known CAD is particularly problematic as patients with more cardiovascular risk factors would be expected to benefit most from statin therapy. These data are consistent with other studies, in which adherence to statin therapy in minority populations has been reported to be lower than the general population, although it should be noted that AIs are underrepresented in these studies.^11,12^ The reason for less statin usage in AIs 1 year after coronary revascularization is unclear but deserves attention as correcting this disparity would be expected to improve survival. For some patients, cost of statins may be a factor depending on the source of their prescription coverage. While generic statins are available, the difference in cost compared to aspirin and beta-blockers may be a barrier and is an area for further study. Certainly, statin therapy is cost-effective and efforts to maintain use, comparable to the general population within the AI population, are warranted.

Most AI patients receive medical care close to home in rural settings where access to health care providers can be problematic. In addition, many patients receive care through the IHSs; federal funding for IHS is lower compared to federal funding per person for other health care programs and this potentially could affect prescribing patterns. Finally, in our environment, care immediately after CABG occurs at the tertiary facility, and a year after surgery, this access to tertiary care services may be more difficult as IHS provider access and IHS access to diagnostic imaging and specialty centers is inadequate.^13^ We speculate that improving coordination and communication between the tertiary health care environment and local providers and healers is likely to help adherence to GDMT, improve patient survival, and improve health maintenance in this higher risk population.

In our study, AIs were more likely to have a prescription for opioids at the time of discharge and were more likely to be on opioids 1 year after surgery than non-AIs. This observation is concerning. The United States is experiencing an opioid epidemic with deaths due to opioid abuse; in 2016, it was five times higher than that in 1999, and AI mortality rates due to opioid overdose have been steadily increasing.^14^ In Minnesota, a state serviced by our tertiary care center, opioid overdose occurred in 47.6 deaths per 100,000 in AIs compared with 7.3 per 100,000 in non-Hispanic whites.^15^ Surgical specialties prescribe more opioids than most other specialties, and perioperative opioid use after surgical procedures has been shown to influence opioid use in the long-term.^16–18^ While postoperative pain control is important, attention to weaning from opioids and alternative pain management strategies in the weeks after CABG is imperative for all patients. In addition, we do not have data regarding preoperative opioid use in our patients, which may be relevant as the indication for opioid use a year after surgery is unclear. The reason for disparities in opioid prescribing patterns between AI and non-AI patients after CABG is an important topic which mandates further investigation

Hypertensive and diabetic medications were also different between AIs and the general population after CABG. AIs were more likely to be on multiple antihypertensive medications 1 year after CABG and specifically more likely to be prescribed ACE-inhibitors and ARBs. ACE-inhibitors and ARBs not only help treat hypertension but also have cardio-protective qualities that are beneficial postoperatively.^19^ The higher incidence of ACE-inhibitors and ARBs may reflect appropriate treatment of the increased cardiovascular risks facing AI patients. Diabetic patients are at especially high cardiovascular risk and the use of ACE-inhibitors and ARBs may help prevent diabetic nephropathy and other long-term complications of diabetes. Blood pressure control is frequently suboptimal in the preoperative and postoperative CABG patient, highlighting the need for better blood pressure control with the use of multiple medications such as beta blockers and ACE-inhibitors/ARBs.

In our study, AI patients were more likely to be discharged and maintained on insulin therapy (Tables 2 and 3) after surgery. In addition, fewer oral hypoglycemics were used 1 year after CABG. Whether this reflects the severity of the disease or changes in prescribing patterns is unclear. AI patients were more likely to present on insulin therapy.^7^ In the immediate perioperative period, insulin therapy (frequently as intravenous infusion) is used as blood sugar fluctuations are quite common. As patients return to their local providers after surgery, an expectation would be for increasing oral hypoglycemic use as the stress of surgery diminishes. Our finding that oral hypoglycemic medication use is reduced in the AI population is consistent with earlier studies showing less effective control and reduced hypoglycemic use in AIs with diabetes.^20^ Whether this is driven by cost is unclear but a potential cause for this disparity, especially for AI patients with inadequate prescription drug coverage.

Formal CR is recommended for all CABG patients postoperatively and has been shown to improve long-term outcomes after CABG, with a 26% risk reduction in the rate of cardiovascular mortality.^8,21^ Furthermore, an inverse dose–response relationship exists between the number of CR sessions attended and long-term rates of myocardial infarction and death.^22^ Our study found that fewer AIs attended formal CR than non-Hispanic whites, although this was not statistically significant. Access to CR may be more difficult for AIs because of cost and lack of third-party payment coverage. While AI patients after CABG may value home healers and caregivers for spiritual or family reasons, combining the benefits of CR with the spiritual well-being of home care is an opportunity for improvement in the prevention of future cardiac events after CABG.^23^ In our treatment area, there is only one tribally run nursing home facility. Increased funding to allow for more tribally run rehabilitation is likely to improve adherence to CR and improve the prevention of CVD.

## Limitations and Summary

This was a single-center, retrospective study of a Northern Plains AI population undergoing CABG and is likely underpowered to detect differences in outcomes and treatments. The AI population of Northern Plains Indians also may not be generalizable to other AI/Alaskan Native populations as cultural, geographic, and socioeconomic differences exist between different tribal regions. However, AIs are an understudied population and little data exist after CABG. Prescribing patterns and medical care also varies regionally in the general population, as does access to tertiary care facilities.

This study highlights an opportunity to improve adherence to guideline-directed medical therapy after CABG, reduce the recurrence of ischemic heart disease, and potentially improve long-term outcomes. While AIs may undergo CABG with excellent short-term outcomes, we identified disparities in medication and rehabilitation therapies that exist between AIs and non-AIs 1 year after surgery. Specifically, increasing statin use, improving CR, smoking cessation, and improving diabetic and hypertension management would be expected to improve long-term outcomes and are an important area for further investigation. Incorporating GDMT within the context of the local tribal environment and traditional healing is critical for acceptance. Finally, while not related to the prevention of ischemic heart disease, differences in prescribing patterns of opioids between AIs and non-AIs are troubling and obviously impactful to AI health. Further investigation into the source of this disparity is indicated.

## Abbreviations Used

ACE: angiotensin converting enzyme
ACEI: angiotensin converting enzyme inhibitor
AI: American Indian
ARBs: angiotensin II receptor blocker
CABG: coronary artery bypass grafting
CAD: coronary artery disease
CR: cardiac rehabilitation
CVD: cardiovascular disease
GDMT: guideline-directed medical therapy
IHS: Indian Health Service
NPH: neutral protamine Hagedorn
NS: not significant
STS: Society of Thoracic Surgeons
